# Lhermitte-Duclos disease with excessive calcification in a septuagenarian: A case report

**DOI:** 10.1097/MD.0000000000036212

**Published:** 2024-01-05

**Authors:** Yang Su, Seidu A. Richard, Zhigang Lan, Yuekang Zhang

**Affiliations:** a Department of Neurosurgery, Tibet Chengban Branch of West China Hospital, Sichuan University, Sichuan, P. R. China; b Department of Neurosurgery, West China Hospital, Sichuan University, Chengdu, P. R. China; c Institute of Neuroscience, Third Affiliated Hospital, Zhengzhou University, Zhengzhou, P. R. China.

**Keywords:** age-related, calcification, cerebellar, Lhermitte-Duclos, septuagenarian, tiger-striped

## Abstract

**Rationale:**

Lhermitte-Duclos disease (LDD), or dysplastic cerebellar gangliocytoma (DCG), is a rare tumor originating from the cerebellar cortex. LDD is a benign neuroglial tumor with uncertain prognosis. Over 200 cases have been reported in the literature mostly in the form of case reports. Thus, we present a spectacular case of LDD with excessive calcification in a female septuagenarian.

**Patient concerns:**

A 72-year-old female presented with progressive dizziness for 8 months and suffered a head and sacrococcygeal region injury 20 days prior to her admission in our neurosurgery department.

**Diagnosis:**

Computed tomography scan showed a right nonspecific cerebellar mass with striated calcification. Magnetic resonance imaging revealed a right “tiger-striped” alteration of the cerebellar cortex. H&E staining revealed a low grade glial neural tumor which was consistent with the diagnosis of LDD or DCG.

**Intervention:**

The lesion was total resected.

**Outcomes:**

The patient recovered well and the cerebellar dysfunctional symptoms subsided 3 months after the operation and 2 years follow-up revealed no recurrence of the lesion and no neurological deficits.

**Lesion:**

We postulate that the calcification of LDD is age-related and the pathogenesis of disease often observed in young adulthood.

## 1. Introduction

Lhermitte-Duclos disease (LDD), or dysplastic cerebellar gangliocytoma (DCG), is a rare tumor originating from the cerebellar cortex mostly in the third or fourth decade of life in both sexes.^[1,2]^ Notably, LDD is a benign neuroglial tumor with uncertain prognosis.^[3]^ The disease is currently classified as World Health Organization grade 1 tumor and under the category of glioneuronal and neuronal tumors in the current classification of central nervous system tumors.^[3]^ Clinically, LDD is depicted with cerebellar dysfunction, hydrocephalus as well as signs of increased intracranial pressure because it causes mass effect in the posterior cranial fossa.^[2]^ The gold-standard radiological diagnostic modality for the detection of this rare disuse entity is the Magnetic resonance imaging (MRI).^[4,5]^ The lesion is often seen with classic “tiger-striped” appearance on MRI.^[5,6]^

However, in cases of excessive calcification, the disease is very apparent on computed tomography (CT) scan imaging as seen in our index case. The disease is typically single and unilateral, presenting as a distinct region of cerebellar hypertrophy.^[3]^ The best therapeutic modality for the treatment of this disease is surgical intervention.^[7,8]^ Pathologically, LDD has been identified as an entity of the phosphatase and tensin homolog (PTEN) hamartomatous tumor syndromes (PHTS) and it is characterized with the absence of the Purkinje cell layer as well as progressive hypertrophy of the granular cell neurons with increased myelination.^[3,4,6]^ We present a spectacular case of LDD with excessive calcification in a female septuagenarian.

## 2. Case report

A 72-year-old female presented with progressive dizziness for 8 months. She accidentally fell down and injury her head and sacrococcygeal region 20 days prior to her admission in our neurosurgery department. She denied headaches, nausea and vomiting, blurring of vision, convulsion, vertigo, ataxia and abnormal behaviors. She is hypertensive and on medications for the past 4 years. All her essential vitals recorded were normal and general physical examination did not yield much. Her pupils were equal in size and sensitive to light reflex. Also, ear, nose and throat examination did not yield much. However, neurological examination revealed dystaxia and dysmetria. Sensory examination and power in both upper and lower limbs were 5/5 with normal Brudzinski and kenigs signs.

**Figure 1.**
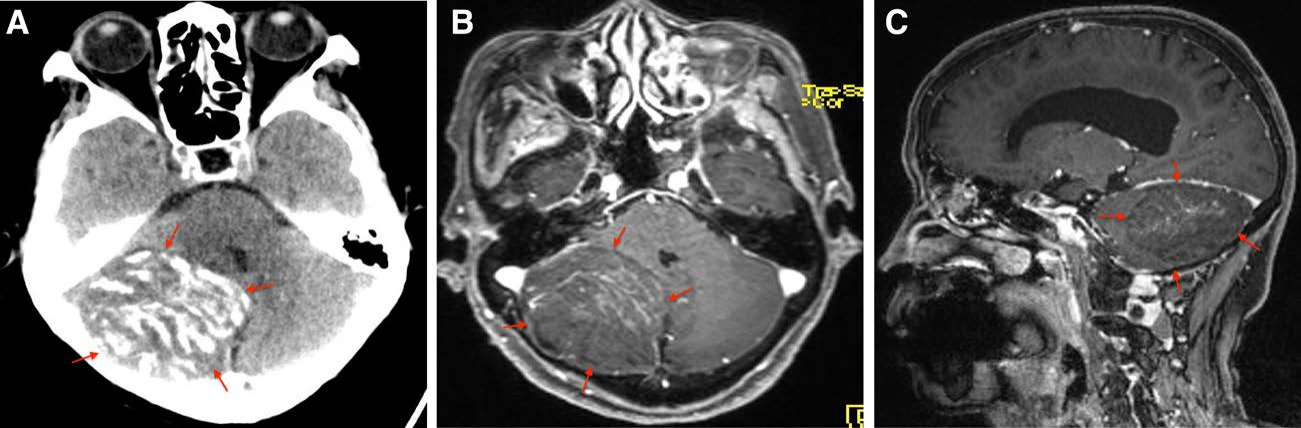
A-C: Are preoperative CT scan and MRIs showing lesion. A = CT scan, B = Axial, C = Sagittal. Red arrows = lesion.

Also, routine laboratory investigations were unremarkable and Chest X-ray as well as electrocardiogram were normal. CT scan showed a right nonspecific hypoattenuating cerebellar mass with striated calcification (Fig. 1A) in the county hospital necessitating her referral to our neurosurgery unit. Also, CT scan showed compressed and deformation of the fourth ventricles with extravasation of cerebrospinal fluid. MRI revealed a right “tiger-striped” alteration of the cerebellar cortex measuring 6.0 × 6.0 × 6.2 cm in the right cerebellum hemisphere (Fig. 1B and C). The striated mass was hypointensity on T1-weighted images and hyperintensity on T2- weighted images interspersing with isointense bands of tissue. They both showed enhancing and non-enhancing areas. Thus, radiological diagnosis of LDD or DCG was made and she was scheduled for surgery.

**Figure 2.**
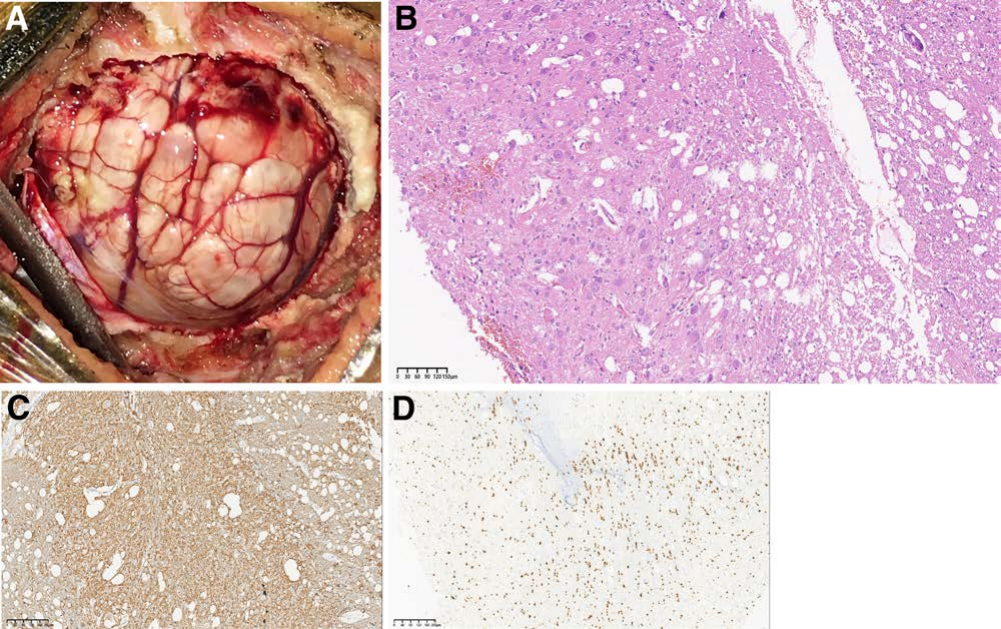
Are intraoperative and pathological images confirming the diagnosis of LDD or DCG. A: Intraoperative image showing the lesion. B: H&E staining showing a low grade glial neural tumor. C: Immunohistochemistry image showing positivity for synaptospysin. D: Immunohistochemistry image showing positivity for OLIG-2.

The patient was put in the park bench position with her head fixed in Mayfield 3 keys’ head support system after the general anesthesia. Routine electromyographic as well as auditory brainstem responses were utilized to monitor the cranial nerves. Intraoperatively, we saw a heavily calcified right cerebellum hemisphere lesion (Fig. 2A). The lesion was composed of solid and soft portions, red and white in color with scanty blood supply and unclear boundaries with the surrounding cerebellar tissues. We achieved total resection of the lesion and repaired the sinus with no neurological deficits. H&E staining revealed a low grade glial neural tumor which was consistent with the diagnosis of LDD or DCG (Fig. 2B).

**Figure 3.**
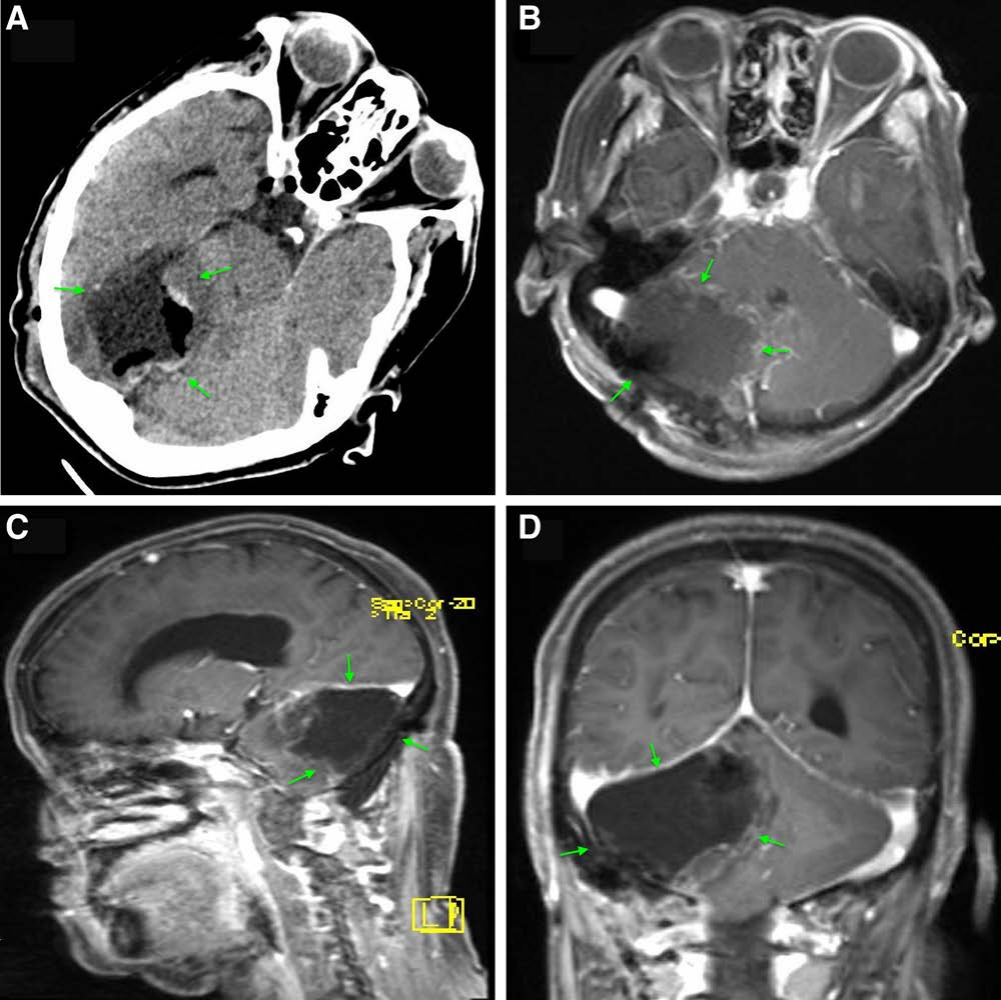
A-D: Are postoperative CT scan and MRIs showing total resection of the lesion. A = CT scan, B = Axial, C = Sagittal, D = Coronal. Green arrows = site of resected lesion.

Immunohistochemical staining revealed positivity (Fig. 2C and D) for NeuN, glial fibrillary acidic protein, OLIGO2, synaptospysin, S-100 and no deletion in ATRX with Ki-67 (MIB) index of 2%. However, p52 was negative. Notably, no codon 132 mutation was detected in IDH1 gene and no codon of 172 mutation was detected in the IDH2 gene too. Also, no mutation was detected in exon 15(V600E or V600K) following BRAF gene mutation analysis. Her postoperative cause was uneventful with no neurological deficits. Postoperative CT scan (Fig. 3A) and MRI revealed total resection of the lesion (Figs. 3B–D). She was discharged home 2 weeks after the operation. She recovered well and the cerebellar dysfunctions symptoms subsided 3 months after the operation. Two years follow-up revealed no recurrence of the lesion and no neurological deficits.

## 3. Discussion

LDD or DCG is a rare disease with uncertain pathogenesis and prognosis.^[3,9]^ This disease classically presents in young adults, although cases have been reported in all ages as well as in both sexes.^[3,10]^ Our case is much more spectacular because of the advance age of the patient and excessive calcification of the lesion. Thus, we speculate that the calcification in our index case is age related. Notably, over 200 cases have been reported in the literature and neoplastic, dysplastic or hamartomatous, have been implicated as the origin of this rare disease.^[2,8]^ Interestingly, most LDD patients appear to have a germline loss of the PTEN allele and progressively lose the remaining PTEN allele at some point resulting in anomalous growth of the granule cells.^[11]^

The PTEN is a tumor suppressor and its mutation typically stimulates overgrowth of tissue.^[10,12]^ Notably, mutations in the genes encoding diverse proteins in the AKT/PI3K signaling pathway triggers a gain of function of the gene products leading to a hamartomatous tumor syndromes such as the Cowden syndrome, Bannayan-Riley-Ruvalcaba syndrome, and Proteus syndrome.^[10,12]^ The clinical presentation varies from asymptomatic to symptomatology such as headache, vertigo, ataxia, cranial nerve palsy as well as psychiatric manifestations.^[3,6,10,13]^ In severe cases, signs and symptoms of intracranial hypertension secondary to hydrocephalus may manifest.^[2,6]^ Mostly, patients have long-standing symptomatology, signifying the slowly progressive pathogenesis of the disease.^[6]^ Our patient’s disease course was progressive and asymptomatic for a long time. However, her key symptomatology was progressive dizziness, dystaxia and dysmetria which result in accidental fall with head and sacrococcygeal region injuries.

LDD typica present on CT, as a hypodense cerebellar mass, without contrast enhancement.^[2,5,6]^ In younger patients, CT is of a limited value, and the only diagnostic signs may be the mass effect, which manifests as compression of the fourth ventricle, effacement of the cerebellopontine angle cistern, as well as hydrocephalus.^[6,11]^ However, in advance age, the lesion presents with excessive “tiger-striped” calcifications in the cerebellum as seen in our index case. Particularly, there is usually a striated display of hypointensity on T1-weighted images as well as a hyperintensity on T2- weighted images interspersing with isointense bands of tissue on MRI.^[4–6]^ Notably, a “tiger-striped” cerebellar lesion with unilateral hemispheric expansion as well as conservation of the gyral configuration is a precise sign for the disease, and these observations is enough for a definitive diagnosis.^[2,4–6]^

In our case, MRI further revealed the “tiger-striped” alteration of the cerebellar cortex the right cerebellum hemisphere. Interestingly, positron emission tomography (PET) as well as single-photon emission computed tomography via radiotracers such as fluorine-18-fluorodeoxyglucose, thallium-201 and C11-methionine has been used for the metabolic assessment of LDD.^[2,5,8]^ Precisely, amalgamation of 18F-FDOPA PET/MRI fusion offered anatomic localization of tracer uptake as well as well-labeled enhancing and non-enhancing tumor areas as well.^[2,14]^ Furthermore, 18F-FDOPA activity detected tumor not visible on MRI in a small number of patients.^[14]^ We did not utilize any of the above radiological modalities because CT scan accurately revealed the diagnosis of tumor.

Surgery is the gold-standard treatment modality for LDD although conservative management is also accepted in cases with minimal symptomatology’s.^[7,8,15,16]^ Remarkably, the lesion often slow growing in nature with unclear boundaries with the adjacent cerebellum making total resection difficult, if not impossible.^[16,17]^ Nevertheless, total resection of the tumor is still considered the most anticipated surgical strategy.^[16,18]^ However, some authors advocate both maximal safe resection as well as preventive C1 laminectomy.^[17]^ Interestingly, intraoperative ultrasound has been used to determine the exact location, specified the lesion resection range, as well as analyzed the blood supply and the venous sinus patency aiding in accurate resection of the lesion.^[17]^ We attained total resection of the lesion in our case with no neurological deficits.

Markedly, subtotal or partial resection of the lesion of lesion was associated with postoperative recurrence in case.^[16]^ Radiotherapy has also previously been used to treat patients with this rare disease.^[15]^ Medically, rapamycin therapy has also been considered in a pediatric patient with success because the disease is considered PHTS.^[11]^It is worth noting that rapamycin inhibits mechanistic target of rapamycin, which is accountable for the stimulation of cell growth as well as proliferation.^[19]^ Our patient recovered well and the cerebellar dysfunctional symptoms subsided 3 months after the operation and 2 years follow-up revealed no recurrence of the lesion and no neurological deficits.

LDD or DCG is depicted with regional enlargement of the cerebellar stratum granulosum, deficient of Purkinje cell layer as well as progressive hypertrophy of the granular cell neurons with augmented myelination on histopathology.^[2,6]^ Also, disorganization of the normal laminar cellular architecture of the cerebellum is usually present. Our H&E staining revealed a low grade glial neural tumor which was consistent with the diagnosis of LDD or DCG. Also, Immunohistochemical staining revealed positivity for NeuN, glial fibrillary acidic protein, OLIGO2, synaptospysin, S-100 and no deletion in ATRX with Ki-67 (MIB) index of 2%. However, p52 was negative. Markedly, no codon 132 mutation was detected in IDH1 gene and no codon of 172 mutation was detected in the IDH2 gene too. Furthermore, no mutation was detected in exon 15(V600E or V600K) following BRAF gene mutation analysis.

## 4. Conclusion

The calcification of LDD is age-related and the pathogenesis of disease often observed in young adulthood. Thus, the disease course can be progressive and asymptomatic for a long time. CT scan accurately establish the diagnosis of tumor in elderly patients. However, it may not be accurate in younger patients.

## Author contributions

**Conceptualization:** Yang Su, Seidu A. Richard, Zhigang Lan, Yuekang Zhang.

**Data curation:** Yang Su, Seidu A. Richard, Zhigang Lan, Yuekang Zhang.

**Formal analysis:** Yang Su, Seidu A. Richard, Zhigang Lan, Yuekang Zhang.

**Funding acquisition:** Zhigang Lan.

**Investigation:** Yang Su, Seidu A. Richard, Zhigang Lan, Yuekang Zhang.

**Methodology:** Yang Su, Seidu A. Richard, Zhigang Lan, Yuekang Zhang.

**Supervision:** Zhigang Lan, Yuekang Zhang.

**Writing – original draft:** Seidu A. Richard.

**Writing – review & editing:** Yang Su, Seidu A. Richard, Zhigang Lan, Yuekang Zhang.
